# Camelina (*Camelina sativa* (L.) Crantz) as Feedstuffs in Meat Type Poultry Diet: A Source of Protein and n-3 Fatty Acids

**DOI:** 10.3390/ani12030295

**Published:** 2022-01-25

**Authors:** Robertas Juodka, Rasa Nainienė, Violeta Juškienė, Remigijus Juška, Raimondas Leikus, Gitana Kadžienė, Daiva Stankevičienė

**Affiliations:** 1 Department of Ecology, Animal Science Institute, Lithuanian University of Health Sciences, R. Zebenkos 12, 82317 Baisogala, Lithuania; violeta.juskiene@lsmuni.lt (V.J.); remigijus.juska@lsmuni.lt (R.J.); gitana.kadziene@lsmuni.lt (G.K.); daiva.stankeviciene@lsmuni.lt (D.S.); 2 Department of Animal Breeding and Reproduction, Animal Science Institute, Lithuanian University of Health Sciences, R. Zebenkos 12, 82317 Baisogala, Lithuania; 3 Department of Animal Feeding and Feedstuffs, Animal Science Institute, Lithuanian University of Health Sciences, R. Zebenkos 12, 82317 Baisogala, Lithuania; raimondas.leikus@lsmuni.lt

**Keywords:** camelina, poultry, n-3 fatty acids, meat quality, growth performance

## Abstract

**Simple Summary:**

One of the main problems in poultry production is to find more sustainable feed protein sources, other than the most widely used soya bean meal. An alternative protein source could be the underexploited oilseed crop camelina (*Camelina sativa* (L.) Crantz), which is mostly grown for biodiesel production, but is also characterized by disease and pest resistance, tolerance to cold weather, drought and low fertility soil. This review presents the nutritive value of camelina seeds, oil and cake (a by-product of biodiesel production), and their effect on the growth performance and fatty acid profile of muscles and liver in meat type poultry. The research results indicated that supplementation of poultry diets with camelina feedstuffs beneficially modified the fatty acid composition of meat and liver. The ratio of n-6/n-3 polyunsaturated fatty acids (PUFA) decreased, whereas the content of α-linolenic and long-chain n-3 PUFA increased in poultry tissues.

**Abstract:**

Camelina seed or seed processing derivatives, i.e., cake, are cheap alternative protein feed ingredients for meat type poultry. Camelina is an oilseed crop containing 36.8% oil in seeds, while in the cake the oil content accounts for 6.4–22.7%. If compared with other *Brassicaceae* family plants, camelina is distinguished by a unique fatty acid composition, because the content of α-linolenic fatty acid (C18:3n-3; ALA) varies from 25.9 to 36.7% of total fatty acids. The total tocopherol content in camelina oil and cake are, respectively, 751–900 and 687 mg/kg. Addition of camelina to poultry nutrition increases the amount of n-3 polyunsaturated fatty acids (PUFA) in poultry meat and liver. The content of ALA in chicken muscles increases by 1.3–4.4, 2.4–2.9 and 2.3–7.2 times after supplementing chicken diets with, respectively, camelina cake (8–24%), seed (10%), and oil (2.5–6.9%) in comparison with the control group. Camelina cake (5–25%), seed (10%) and oil (2.5–4%) inclusion in chicken diets results in 1.5–3.9 times higher total n-3 PUFA content in muscles and liver. Meanwhile, supplementation of chicken diets with camelina oil (4–6.9%), seed (5–10%) and cake (5–25%) results in, respectively, a 1.8–8.4, 1.6–1.9 and 1.3–2.9 times lower n-6/n-3 PUFA ratio in muscles, and 3.29 times lower n-6/n-3 PUFA ratio in the liver. After inclusion of different amounts of camelina cake in chicken diets, a healthy for human nutrition n-6/n-3 PUFA ratio from 1.6 to 2.9 was found in chicken muscles.

## 1. Introduction

Modern animal farming systems are meant to solve global challenges such as providing the growing human population with safe food, and mitigating environment pollution and climate change. This can be achieved by more efficient use of natural resources, minimizing greenhouse gas emissions and becoming more resilient against climate change.

The requirements to reduce the environmental and climate impact of animal production and transfer to more sustainable livestock production are provided for in the EU Farm to Fork Strategy [1]. About 75% of protein feeds required for the balance of livestock feed rations is imported into the EU [2]. Thus, the sustainable production strategy can be implemented by reducing the dependency on critical feed materials, for example, soya, which is grown on deforested land. One of the ways to solve the problem would be growing alternative protein crop varieties that are well-adapted to certain rural area conditions, require less fertilizers and are resistant to diseases and agricultural pests.

Currently, human nutrition is deficient of n-3 polyunsaturated fatty acids (PUFA). Epidemiologic studies indicate that such deficiency could be the cause of coronary heart disease, cancer and depression [3,4]. One of the solutions to the problem could be poultry production with increased n-3 PUFA content by supplementing poultry with diets rich in n-3 PUFA feeds as, for example, camelina seed, cake or oil [5,6,7,8,9].

Camelina (*Camelina sativa* (L.) Crantz) is a protein and oilseed crop belonging to the family *Brassicaceae* [8]. Numerous archaeobotanical studies indicate that camelina has a long history of its cultivation in Europe and Asia Minor [10,11,12,13]. In many European countries camelina was grown as an agricultural crop until the mid-20th century [14]. 

Camelina has many positive attributes [15]. When compared with conventional oilseed crops, such as rape and sunflower, camelina possesses considerable agrotechnical and industrial benefits [14]. The crop can be grown under different climatic conditions and with a low input, as it is tolerant to drought, low temperature and heat [16,17,18]. Due to lower demand for nutrients and water, it can be cultivated with no irrigation and a lower fertilization level, and often on marginal and saline soils [19,20]. Camelina is more resistant to many pests and diseases than other *Brassicaceae* family plants and, therefore, its cultivation is more environmentally friendly due to the reduced use of herbicides and pesticides [14,21,22,23]. 

Camelina is not widely grown in the world. The main producers are in North America, Europe and some other parts of the world. In the USA and Canada, it covers an area of several thousand hectares; in the Russian Federation it covers 75.9 thousand hectares; in Lithuania it ranges from 23.8 to 82.7 hectares [24,25,26]. Camelina productivity is highly affected by climate conditions and varies widely [26]. The seed yield in Germany is about 1.9 t/ha, France—2.8 t/ha, Poland—1.75 t/ha, USA—2.3 t/ha, Russia—0.69 t/ha, Lithuania—from 0. 8 to 2.1 t/ha [24,25,26,27]. The seed yields can reach up to 1.1 t/ha even under conditions of limiting nutrients or water. Under favorable conditions, camelina crops yield more than 3 t/ha [26]. However, the productivity of camelina is almost twice as low as that of rapeseed.

Camelina seed contains a large amount (36.8%) of oil characterized by a unique composition of fatty acids, i.e., a high content of n-3 PUFA with α-linolenic (C18:3n-3; ALA), accounting for 25.88–36.67%.

Camelina has recently attracted great interest as an oil crop for biodiesel, jet fuel and oil production with a low production cost [28,29,30]. Large quantities of by-products (cake, meal) are left after oil extraction. Camelina expeller, or cake, is a product of oil extraction obtained by pressing camelina seed [31]. Cake contains from 6.4 to 22.7% residual oil (Table 1).

These products have good potential to be used as a cheap alternative protein feedstuff and a valuable source in animal nutrition [14,31,34,35]. Camelina by-products are comparatively cheaper than other sources, especially soybean [31]. 

The objective of this review is to discuss chemical, amino and fatty acid composition of various feed ingredients produced from camelina and their effects on meat type poultry growth performance, carcass traits, meat chemical composition and tissue fatty acid profile.

## 2. Camelina Chemical Composition

Camelina is a valuable source of protein. Different camelina feed components contain different amounts of protein. Camelina seed contains 24.78% protein, while seed by-products, such as cake, have higher protein content, respectively, 30.33–39.80% (Table 1).

The crude protein content in camelina cake is close to that found in rapeseed meal (29.69–39.89%), but lower than in soybean meal (43.0–56.3%) [33,34,37,38,39]. 

The crude fat content found in camelina seed is high (36.84%). However, the crude fat content in cake is in the range of 6.44–22.71% (Table 1). The crude fat content in camelina cake is higher than in soybean (0.55–3.3%), and rapeseed (1.4–10.50%) meals [33,34,36,37,38,39,40,41,42]. 

High crude fiber content is found in camelina seed by-products in the range of 9.7–17.40% (Table 1). The crude fiber content in camelina by-products is higher than in soybean meal and similar to that in rapeseed meal and expellers [8,15,33,34,43].

Carbohydrates of camelina seed include monosaccharides, disaccharides, oligosaccharides and polysaccharides. The content of sucrose in camelina seed is the highest among carbohydrates and accounts for 5.5%. The content of starch and pectin (polysaccharides) are 1.21 and 0.96%, respectively [44]. The lignin content in camelina seed is 7.4% [44]. The content of mucilage in camelina is 6.7%, and it is lower than in flaxseed (8%) [44,45].

### 2.1. Amino Acid Composition in Camelina

It is known that amino acid composition, especially essential, shows the biological value of protein. The essential amino acids in camelina cake are in the range of 15.09–18.39% (Table 2).

Methionine with cystine and lysine are the first limiting acids in poultry nutrition. The content of methionine with cystine (3.80–5.17% of total protein) in camelina cake is higher than soybean meal (2.61–3.27% of total protein) and similar to that in rapeseed meal (4.12–5.01% of total protein). The content of lysine in camelina is 1.55–2.02 and 1.02–1.56 times lower than that in soybean meal and rapeseed meal, respectively [8,33,39,46,47,48,49,50]. 

The content of arginine, valine, alanine and proline in camelina are lower than in soybean meal, but by total protein content, the difference is insignificant [8,33,39,47,49,50]. In comparison with rapeseed meal, camelina cake has a similar content of alanine, asparagine, glycine, isoleucine, leucine, phenylalanine, serine, tryptophan, tyrosine and valine [8,33,46,47,48,49,50]. 

The above data conclude that camelina cake is very close to rapeseed meal in terms of its amino acid composition, and is a valuable raw material in poultry feeding.

### 2.2. Camelina Fatty Acid Composition

The amount of total saturated fatty acids (SFA) in camelina cake and oil (Table 3) is lower than in soybean meal (19.94%) and rapeseed cake (16.30%), but higher than in hempseed cake (7.66%) [51,52]. 

The amount of total monounsaturated fatty acids (MUFA) in camelina cake and oil is higher than in hempseed cake (10%), but lower than in soybean meal (52.43%) and rapeseed cake (48.66%) [51,52]. The content of harmful erucic (C22: 1n-9) MUFA in camelina cake is 35.6 times higher than rapeseed cake [52]. 

The percentage of highly important n-3 ALA is usually low in the main feed ingredients of plant origin. Thus, the amount of ALA usually found in wheat is 0.06–0.14% [54], in corn 0.48–0.50% [55], in sunflower 0.15–0.27% [56] and in barley 0.35% [53]. 

Camelina seed, oil and its processing products have a much higher ALA content (25.88–36.67%) than other common feed components, i.e., soybean meal (7.21–8.58%), hempseed cake (15.85–24.7%), and rapeseed cake (10.60–13.05%), but lower than that found in linseed cake (51.5%) [51,52,57,58]. Consequently, it may be maintained that camelina is the second highest, by ALA content, plant growing in the northern hemisphere, and suitable as a feed component.

Essential n-6 PUFA linoleic (C18:2n-6; LA) fatty acid content, which should be as low as possible in poultry feed, amounts to 18.84–24.16% in camelina seed and cake, and this percentage is lower than that found in rapeseed cake (21.67–23.5%), soybean meal (52.43–55.20%) and hempseed cake (52.5–59.52%), but higher than that found in linseed cake (14.60%) [15,34,51,52,53,58,59,60,61,62]. 

The content of total n-3 PUFA, which is always deficient in standard poultry feeding, is from 3.11 to 4.00, 2.05 to 3.96 and 1.0 to 2.16 times higher in camelina cake and seed, than that found in soybean meal, rapeseed cake and hempseed cake, respectively, but from 1.50 to 2.19 times lower than in linseed cake [50,52,59,60]. Camelina cake and oil has a lower n-6/n-3 PUFA ratio in comparison with soybean meal, rapeseed cake and hempseed cake, respectively, 0.60–0.99 vs. 6.10, 1.68, 4.08 [37,51,52]. The linoleic/α-linolenic ratio in camelina cake (0.72) was also lower compared with rapeseed cake (1.66), hempseed cake (3.76) and soybean meal (6.11) [51,52,53]. 

### 2.3. Camelina Vitamins, Macroelements and Microelements

The content of vitamin B_3_ (niacin) in camelina seed (194 µg/g) is predominant among the vitamins, with camelina seed containing about twice the amount as occurring in flaxseed (91 μg/g) [44]. The content of vitamin B_1_ (thiamin) and vitamin B_5_ (pantothenic acid) in camelina are 18 μg/g and 11.3 μg/g, respectively [44]. The content of thiamin in camelina is considerably higher in comparison with flaxseed (6 μg/g) and rapeseed (8 μg/g) [44]. 

The content of pantothenic acid is identical to flaxseed (11 μg/g) and lower than rapeseed (16 μg/g) [44].

The content of other B group vitamins is low, i.e., B_2_ (riboflavin) 4.4 μg/g, B_9_ (folate) 3.2 μg/g, B_6_ (pyridoxine) 1.9 μg/g, and B_7_ (biotin) 1.0 μg/g [44]. 

Camelina seed contains macro-minerals in small amounts. The highest amounts are those of potassium (K), phosphorus (P), and calcium (Ca), being respectively, 1.6, 1.4 and 1.0% [44]. Zubr [44] has also reported small amounts of magnesium (0.51%), sulfur (0.24%), sodium (0.06%) and chlorine (0.04%) in camelina seed. Among micro-minerals, camelina seed has a remarkably high content of iron (329 μg/g) with substantial content of manganese (40 μg/g) and zinc (69 μg/g) [44]. The copper content is 9.9 μg/g, and nickel content is 1.9 μg/g [44]. 

### 2.4. Antioxidant Content in Camelina

Camelina oil contains high levels of γ-tocopherol (710 mg/kg [62]. Other tocopherols are α-tocopherol (28.07–41.8 mg/kg) and δ-tocopherol (12.3–20.47 mg/kg) [63]. 

The total tocopherol content determined in fresh camelina oil amounted from 751 to 687 mg/kg and this amount was higher than in flax oil and rapeseed oil [63,64]. 

A high level of phenolic acid and flavonoids have also been found in camelina. The content of phenolic acid in camelina seed ranges from 2043.6 to 3704.7 mg/kg, in oil from 681.89 to 892.12 mg/L, and in cake from 1148.67 to 1413.76 mg/kg of dry matter [65,66]. 

The content of flavonoids in camelina seed, oil and cake have been found to be from 329.49 to 526.4 mg/kg, 266.01 to 435.32 mg/L, and 37.69 to 73.13 mg/g, respectively [65,66]. Antioxidants present in camelina prolong the storage time for oil, seeds and cake because they reduce lipid oxidation.

### 2.5. Antinutritive Compounds in Camelina

The use of camelina feedstock in poultry nutrition is limited by plant secondary metabolites, i.e., glucosinolates, sinapine, phytic acid and condensed tannins that are ascribed to antinutritive compounds found in camelina.

Glucosinolates are natural substances found in *Brassicaceae* family plants [67,68]. Currently, over 140 different glucosinolates are known [69]. Glucosinolates are stable and non-toxic when found in intact plant cells. However, during harvest, storage, feed manufacture and chewing by animals, plant cells are damaged, myrosinases are released, and various toxic glucosinolate transformation products are formed, including isothiocyanates, thiocyanates, nitriles, epithionitriles and oxazolidinethiones, which disturb thyroid and liver function [68,69,70]. 

Glucosinolate accumulation in camelina depends on many factors—genotype, climatic conditions, soil type, sulfur content in the soil, and fertilization [14,71]. Therefore, a wide range of glucosinolate content can be found in camelina. The content of glucosinolates in whole seed, as reported by Schuster and Friedt [71], varies from 13.2 to 36.2 μmol/g and the mean value is 24 μmol/g; while Matthäus and Zubr [14] indicated it ranges from 9 to 19 μmol/g. The amount of glucosinolates in camelina cake is from 14.5 to 44.9 μmol/g [14,36,46,72,73,74]. Matthäus and Zubr [14] indicated that glucosinolates are stored in the residue when oil is produced during seed pressing. Research data show that whole seed contains 14.1 μg/mg, seed meal 24.3 μg/mg, and defatted meal as much as 31.8 μg/mg glucosinolates [75].

Camelina seeds contain unique glucosinolates with long aliphatic side chains that are not found in rapeseed. Glucocamelinin (10-methylsulfinyldecyl-Gls) is the main glucosinolate accounting for 62–72% of the total glucosinolates. The other glucosinolates, 9-methylsulfinylnonyl-Gls and 11-methylsulfinylundecyl-Gls, account for, respectively, 30 and 10% of the total glucosinolates [71,76]. It is assumed that glucosinolates with longer side-chains should have a smaller effect [77,78] than short-chain sulfinylglucosinolates, such as glucoiberin. Thus, from the nutritional point of view, the effect of glucosinolates in camelina can be considered smaller than the effect of glucosinolates in rapeseed products [14].

Woyengo et al. [36] indicate that poultry can tolerate up to 2.0 μmol/g glucosinolates in rapeseed diets, while Tripathi and Mishra [68] increase the tolerance level to 5.6 μmol/g. No sufficient research data can be found to define the effects of camelina-specific glucosinolates and their metabolic products on poultry nutrition.

Since the amount of glycosinolates in camelina varieties varies widely, it indicates a high phenotypic variation, which is a prerequisite for successful selection. Currently, the major breeding objectives for camelina are to increase seed yield, seed oil and protein content, and resistance to abiotic stress, however, varieties with low glucosinolate levels have not been developed.

Sinapine is a choline ester of sinapis acid. Accumulating sinapine is typical of plants belonging to the *Brassicaceae* family. The content of sinapine varies markedly in camelina plants. The seed analysis of eight different camelina genotypes indicated the range of sinapine content to be from 2.8 to 7.8 mg/g, with an average of 4 mg/g [76]. Meanwhile, the analysis of 30 camelina cultivars from different European localities showed that sinapine concentration in oilseed cake is from 1.7 to 4.2 g/kg [14]. A similar mean sinapine content is found in camelina cake by other researchers, i.e., 2.32 g/kg [72], 2.57 g/kg [74] and 2.79 g/kg [73]. The content of sinapine in camelina is much lower than that found in other *Brassicaceae* family plants such as rape or mustard (7 and 13 mg/g, respectively) [14]. Feedstuffs with sinapine taste bitter, but as taste buds in birds are poorly developed [79], feed bitterness does not reduce voluntary feed intake in broilers [80]. However, if no more than 10% of camelina cake is used in meat poultry diets, no undesirable sinapine effect will be found, due to a low sinapine concentration.

Phytic acid has a strong antinutritive effect because it binds phosphorus; however, birds, as monogastric animals, have no enzymes to hydrolyze the bound phosphorus [73].

The content of phytic acid in camelina seed was found to be 21 mg/g [81], and in cakes from 21.0 to 32.3 mg/g [14,72,73]. The amount of phytic acid in camelina is similar to that in sunflower but 1.5 times higher than in rapeseed [76]. Recent studies have shown that phytic acid also has a beneficial effect on health due to its antioxidative properties [14]. 

Condensed tannins (flavan-3-ol based biopolymers) are found in all plant seed. The antinutritive effect of these compounds is displayed by protein precipitation, inhibition of digestive enzyme (trypsin and chymotrypsin) activity and, consequently, feed protein digestibility decrease [72]. Tannins also upset the efficient use of vitamins and minerals. They can make complexes with vitamin B_12_ and, thus, reduce its absorption [72]. The average content of condensed tannins amounts to 1.1, and ranges from 1.0 to 2.4 mg/g in, respectively, camelina seed [76] and cake [14]. By the data from 12 camelina genotypes, the tannin amount varies from 1.92 to 4.39 g/kg, with an average content of 3.1 g/kg [72]. The tannin content in the other study was found to be from 1.81 to 2.59 g/kg [73]. The amounts of condensed tannins in camelina are relatively low and, therefore, there is either no, or a very insignificant, negative effect on poultry nutrition, as tannins show their toxicity only at over 1% amount in the diet [82]. Meanwhile, even small amount of tannins might have a positive effect on animal health as tannins possess antimicrobial as well as anticarcinogenic and antimutagenic properties [83].

On the basis of glucosinolate content studies in rapeseed, the European Food Safety Authority recommends the total glucosinolate content to be not higher than 1–1.5 mmol kg^−1^ in the diets of monogastric animals [69]. The US Food and Drug Administration approved inclusion of up to 10% of the weight of the total ration of the diets of beef cattle and poultry [84]. In Canada and the USA, the standard for glucosinolate content in dried canola meal is set at a maximum of 30 μmol/g of dry matter, and in the EU this value should not exceed 20 μmol/g [69,73]. 

Since amount of glucosinolates in camelina varies widely, it is recommended to investigate the glucosinolate content in camelina seed and cake to calculate their inclusion rate in diets.

## 3. Influence of Camelina on Growth Performance

Different dietary camelina components and different amounts of their inclusion showed different effects on the growth performance of poultry (Table 4).

Inclusion of 5% camelina seed in the diet had no effect on the growth performance of chickens, but feeding 10% seed resulted in a lower body weight (BW) and BW gain [15,87].

The amount of dietary camelina oil ranging from 2.5 to 4.07–6.91% also did not have any effect on the BW, BW gain, feed intake (FI) or feed conversion ratio (FCR) of chickens [15,37,85,88]. This could be explained by a lower amount of antinutrients in oil in comparison with cake or seed [75].

The effects of dietary camelina cake on the growth performance of poultry are contradictory. Studies indicate that supplementation of the diets with 8 and 16% camelina cake increased BW and BW gain [35]. 

Other researchers who used from 5 to 10 [7,53,85,90] and 24% [35] cake in broiler chicken diets have not found any differences in the growth performance parameters.

Studies with quail (5 and 10% cake) and turkeys (5% cake) also indicated that dietary camelina had no influence on BW, FI and FCR data [34,86].

However, other authors indicated that camelina cake had a negative effect on the growth performance of poultry. Ryhänen et al. [8] indicated that 5 and 10% camelina cake inclusion in chicken diets resulted in lower BW, FI (days 1–14) and higher FCR.

Supplementation of quail diets with a higher content of camelina cake (15–20%) resulted in a higher FCR [34]. 

Studies also indicated that 15 to 20% camelina cake inclusion in the diets of turkey poults at the first starter phase of up to 4 weeks of age, and 10% cake inclusion in the diets of chickens up to 21 days of age, had a negative effect on BW and FI because chicks and poults do not have a fully developed digestive system and, therefore, lower ability to digest camelina cake [69,86,89].

Many researchers indicated that the reason for poorer growth performance was the presence of antinutrients, the amounts of which were also different in camelina cakes derived from different source materials. It was found that the glucosinolate content in camelina cake could be from 14.5 to 44.9 µmol/g and that might also influence the growth performance results [8,14,35,89]. Toxic glucosinolate transformation products as thiocyanates and oxazolidinethiones disturb the thyroid function, negatively affect growth, fertility and reproduction and reduce feed conversion [70]. Nitriles irritate the gastro-intestinal mucosa and cause local necroses and hepatotoxicity and nephrotoxicity effects [69].

Other authors found that increasing levels of dietary camelina cake from 3 to 15% in broiler chicken diets reduced the apparent total tract digestibility of dry matter, nitrogen and energy [46]. Pekel et al. [89] reported that camelina cake in broiler diets increased viscosity observed in jejunal digesta and, consequently, reduced utilization of energy and nitrogen.

Moreover, 2.24 to 5.44% fiber was found with 5 to 16% camelina cake inclusion in chicken diets, and in many trials, this amount of fiber was higher than recommended (2.41–2.56%) for Ross cross chicken nutrition management [8,35,91]. Non-starch polysaccharides (NSP), which make the basis of fiber, are poorly digested by poultry due to nutrient encapsulating in cell walls [92]. Research shows that when camelina cake amounts to 30%, fiber-degrading enzymes, i.e., carbohydrases, should be used to degrade NSP in order to reduce feed viscosity, improve nutrient utilization and, consequently, poultry growth and FCR [93]. 

Contradictory research data could be explained by different camelina seed qualities due to different growing and climatic conditions, different camelina cake production methods and trial characteristics. Additional investigation in this area is warranted to further clarify the optimum inclusion rate of ingredients produced from camelina in order to achieve steady growth performance results. 

## 4. Influence of Camelina on Anatomical Dissection Data

Camelina cake, seed and oil inclusion in chicken and quail feed had no influence on their anatomical dissection data (Table 5), except for weight decrease in a poultry specific Bursa of Fabricius lymphoid body part at inclusion of 10% seed and 8% cake when, respectively, a 1.65 and 1.38 times lower weight was found [15,53]. The reduction in Bursa of Fabricius can indicate a weakened body immunity and lower resistance to infection. However, the studies with camelina did not show any negative changes in other organs, such as spleen and thymus, which also take part in poultry immunity formation. A higher mortality in the above groups has not been found either. It could be that the level of Bursa of Fabricius reduction was not so significant as to have an effect on poultry health [15,53].

## 5. Influence of Camelina on Chemical Composition of Breast Muscle

The chemical composition of chicken breast muscle is presented in Table 6. Ciurescu et al. [15] indicated that 5 and 10% camelina seed supplementation of chicken feed resulted in 1.04 to 1.08 times higher protein content. Pietras and Orzcewska-Dudek [88] added 3% camelina oil to chicken feed and reported 1.02 times higher protein content in the breast muscle. However, no differences in protein content were found at 4% oil or 10% cake [85], 6% oil [88] or 2.5% oil [15] inclusion in chicken feed.

## 6. Influence of Camelina on Blood Plasma Parameters in Broiler Chicken

Different studies showed that the use of camelina cake (8%), oil (2.5 and 6%), and seed (5 and 10%) decreased the total cholesterol content in blood plasma (Table 7) by 1.13 to 1.25 times [15,53,88]. 

The studies, of the same authors, carried out with the highest amounts of different camelina by-products, resulted in a decrease in low-density lipoprotein cholesterol (LDL). Thus, 8% cake, 10% seed and 6% oil have decreased the content of LDL by 1.41, 1.54 and 1.47 times, respectively [15,53,88]. 

However, lower amounts of seed (5%) and oil (2.5 and 3%) in chicken diets did not affect the content of LDL in blood plasma [15,88]. 

## 7. Influence of Camelina on SFA and MUFA Composition in Breast, Leg Muscles and Liver

The Food and Agriculture Organization of the United Nations (FAO) recommends reducing the amount of SFA for human consumption [94]. Among all SFA, myristic (C14:0) and palmitic (16:0) acids are considered to be the most harmful in human food [95,96].

Supplementation of chicken diets with camelina cake (2.5–24%) reduced the amount of myristic (C14:0) acid in the liver by 1.15–5.14 times (Table 8).

The amount of palmitic (16:0) acid was from 1.02 to 1.16 times lower in chicken breast muscles with 5–25% dietary camelina cake inclusion. A similar reduction from 1.07 to 1.21 times was also found in leg muscles with 5 to 10% dietary cake inclusion [8,97,98]. Nain et al. [97] reported that 16 and 24% cake supplementation of chicken diets resulted in the highest reduction (from 1.08 to 1.25 times) of palmitic (C16:0) acid detected in the liver. 

However, the use of camelina oil (6.91–4.07%) instead of rapeseed oil increased the amount of palmitic acid (16:0) in breast and leg muscles by, respectively, 1.36 and 1.19 times [37]. A tendency for a higher content of palmitic acid (C16:0) was also observed in the trial with ducks fed 15–20% cake [52]. 

Camelina inclusion in poultry diets resulted in different total SFA profile changes in the muscles and liver.

Dietary camelina cake for chickens had a positive effect on the total SFA decrease in the muscles. Inclusion of 16 and 24% cake resulted in 1.07 to 1.09 times lower total SFA content in breast muscles, while 10% cake inclusion showed a 1.11 to 1.17 times lower total SFA content in leg muscles [97].

Aziza et al. [7] indicated that the total SFA content in the liver of chickens was 1.30 times lower at 2.5% cake inclusion in the diet. However, higher content of cake (up to 5–24%) in the diet resulted in a 1.1 times higher total SFA increase in the liver [7,97]. 

Conversely, camelina oil addition to the feed increased the total SFA content by 1.29 and 1.35 times in, respectively, breast and leg muscles [37]. Juodka et al. [52] reported a 1.08 times higher total SFA increase in the leg muscles of ducks fed 15–20% cake.

Studies indicated that supplementation of poultry diets with camelina cake lowered total MUFA content in the tissues. 

Inclusion of camelina cake (8–24%) reduced the total MUFA content in breast muscles and the liver by 1.07 to 1.23, and 1.2 to 2.32 times, respectively [85,97].

Inclusion of 5, 10 and up to 24% cake in chicken or duck diets resulted in, respectively, 1.04 to 1.18, and 1.08 times lower MUFA content in leg muscles [52,97].

The decrease in MUFA in the muscles and liver was mostly influenced by the reduction in oleic acid (C18:1n-9).

It can be concluded that inclusion of camelina cake in chicken diets reduced the content of palmitic (C16:0) fatty acid and the total SFA in muscles. However, the use of camelina oil increased both the content of palmitic (C16:0) fatty acid and that of the total SFA in muscles. The content of MUFA in the muscles and liver was reduced with camelina cake inclusion in poultry diets. 

## 8. Influence of Camelina on PUFA Composition in Breast, Leg Muscles and Liver

Poultry feeding with a standard compound feed results in a low content of the total n-3 PUFA (from 1.63 to 3.88%) and a comparatively high n-6/n-3 PUFA ratio (from 13.22 to 43.3) in breast and leg muscles [7,15,85,99,100,101,102]. The n-6/n-3 PUFA ratio in the liver is also very high (9.50–12.72) [7,103]. This fatty acid composition is not beneficial to human nutrition. 

Therefore, after Nguyen et al. [104] had found linear correlations between the content of PUFA in feeds and in the tissues of monogastric animals offered PUFA-containing diets, researchers conducted numerous trials aiming for the modification of meat fatty acid composition so as to be beneficial to human health. In agreement with the above studies, Kanakri et al. [105] indicated 0.999 Pearson correlations between n-3 PUFA levels in the diets and tissues of meat type chickens.

The diets for chickens and ducks could include different camelina components such as seed (5–10%), cake (2.5–24%) and oil (2.5–6.91%). 

The total n-3 PUFA content in breast and leg muscles could be increased, respectively, from 1.48 to 2.83, and 1.32 to 3.73 times, and in the liver from 1.62 to 3.90 times (Table 9). These changes have been mostly influenced by the increase in one of the main n-3 PUFA ALA in breast and leg muscles (Table 9).

**Figure 1:**
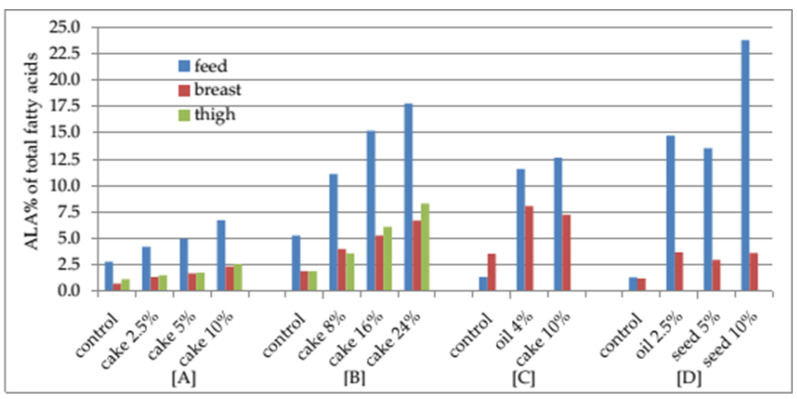
Effect of ALA(%) in diet on ALA(%) in muscles. [A], [B], [C], [D] data presented according to [7,15,85,97].

The least statistically significant ALA increases, by 1.78 and 1.32 times in, respectively, breast and leg muscles, were found after 2.5% cake inclusion in chicken diets (Figure 1). Meanwhile, supplementation of poultry diets with up to 25% camelina cake resulted in 3.9 and 4.37 times higher ALA content in, respectively, breast and leg muscles [97,98]. The highest ALA increases in chicken breast and leg muscles by 7.23 and 6.60 times, respectively, were found after rapeseed oil had been replaced by camelina oil [37]. The studies have indicated that the higher the ALA content was in a poultry diet, the higher was its concentration in the muscles. 

Long-chain (LC) n-3 fatty acids such as eicosapentaenoic (C20:5n-3; EPA) and docosahexaenoic (C22:6n-3; DHA) are very important in human nutrition. However, the conversion of these acids from ALA in adults is lower than 5% [106] and, therefore, human nutrition should be provided with LC n-3 PUFA [107]. Birds are known for considerably more effective synthesis of LC n-3 PUFA from ALA, and, therefore, LC n-3 PUFA accumulation in poultry tissues is preconditioned by ALA being the precursor of all LC n-3 PUFA [107]. 

The increase in eicosatrienoic fatty acid (C20:3n-3; ETE) in chicken and duck muscles and the liver was from 0.08 to 0.37% [52,97]. Meanwhile, camelina seed, oil and cake feeding resulted in 0.15 to 1.08% higher EPA content in breast muscles [15,98] though other researchers did not report any changes with 2.5 to 24% cake addition to the diet [7,52,97]. This is in agreement with the statement by Rymer and Gyvens [108] that there is at best a very weak relationship between dietary ALA content and tissue EPA content.

The amount of another LC n-3 PUFA, docosapentaenoic fatty acid (C22:5n-3; DPA), increased from 1.67 to 2.35, and 1.9 to 3.77 times in, respectively, leg muscles and the liver [97,98]. Meanwhile, the addition of small amounts (2.5–10%) of camelina cake did not increase DPA content in breast muscles [97,98]. However, supplementation of the diets with camelina seed, oil or a larger amount of cake (16–24%) resulted in 1.17 to 1.72 times higher tissue DPA content [15,97].

Bioconversion of dietary ALA is clearly indicated by tissue deposition of ETE and DPA which are transitional metabolites of ALA bioconversion to DHA [97]. 

The effect of camelina components in the feed on the increase in LC DHA in the muscles is controversial. Feeding with 2.5% oil, or 5 and 10% seed, or 5–25% cake resulted in 1.22 to 4.12 times higher DHA content in breast muscles [15,98], whereas DHA increase in leg muscles was from 2.67 to 3.67 times higher than compared with the control group. However, other authors have reported no DHA increase in the muscles when feeding similar amounts of cake (2.5–24%) [97,98]. Rymer and Givens [108] have also indicated that there was no relationship between dietary ALA content and meat DHA content. 

Feeding 5 to 24% cake resulted in 1.39 to 3.31 times higher DHA content in the liver [7,97]. Liver n-3 PUFA profile was distinguished by DHA domination accounting from 29 to 66.44% of total n-3 PUFA [7,97], due to the greater ability of poultry liver to convert dietary ALA to DHA [109].

Studies indicate that the accumulation efficiency of n-3 PUFA was different for the different tissue types [110]. Camelina inclusion in poultry diets showed that the ALA content accounted for 1.35 to 8.07%, and 1.53 to 9.96%, in the fatty acid profile of, respectively, breast and leg muscles. The preferential deposition of ALA in the leg muscles can be explained by the fact that triglycerides are dominant in the intramuscular fat in leg muscles [111]. Meanwhile, DHA tends to be accumulated in phospholipids that are prevalent among breast tissue lipids. Therefore, the DHA content in the breast and leg fatty acid profile accounted for, respectively, 0.66 to 2.35%, and 0.4 to 0.55% [7,8,15,85,97,98,111].

Sensory analyses of cooked meat indicated that camelina had no influence on the organoleptic quality of meat [8,85,88]. No differences were found at evaluation of flavor, tenderness and tastiness. Higher juiciness was reported in one of the studies with camelina oil [85].

The content of n-6 PUFA in camelina cake is comparatively high, but it is lower than that of n-3 PUFA, and the n-6/n-3 ratio is mostly 0.72. The effect of dietary camelina on n-6 PUFA changes in poultry tissues is not clear. The amount of essential n-6 PUFA LA increased from 1.08 to 2.10 times in chicken muscles after a dietary inclusion of 8 to 24% cake, or 6.91 to 4.07% oil [37,85,97].

However, other authors indicated no changes of LA content in breast muscles after poultry diet supplementation with 2.5–4% seed, 4% oil, or 2.5–25% cake [15,52,85,87,98]. No changes of LA content in leg muscles were found with 2.5–20% cake in the diet [7,8,52].

It should be noted that LA accumulation in the muscles is lower than that of ALA, and the reason for this could be the assumption that higher dietary ALA causes competition for the same elongation–desaturation enzymes necessary for the synthesis of both n-3 and n-6 LC fatty acids, thus, resulting in a lower LA content [112].

Studies indicate that one in vivo PUFA metabolism regulating factors is dietary fatty acid composition [110,113,114]. Jing et al. [110] have found that the expression of FADS1, FADS2, ELOVL2 and ELOVL5 genes related with lipid metabolism in the liver of broiler chickens was higher when the linoleic/α-linolenic ratio in the diet was lower. Other studies indicated that ALA content in chicken diets also increased the expression of genes related with lipid metabolism (FADS1, FADS2, ELOVL2, ELOVL5) in breast muscles [115].

Feeding camelina resulted in higher n-3 PUFA amount in muscles and liver and, thus, the n-6/n-3 PUFA ratio decreased and was closer to that suitable for healthy human nutrition [116]. 

Inclusion of 5–25% cake or 4% oil in chicken and 15–20% cake in duck diets resulted in 1.57–2.86 n-6/n-3 ratio in poultry breast muscles [52,85,97,98]. Similar changes were found in chicken and duck leg muscles when n-6/n-3 PUFA ratio decreased and ranged from 1.9 to 2.7 with 8 to 25% dietary camelina cake, or 4.07 to 6.91% oil inclusion [7,8,37,52,97]. In the breast muscles the n-6/n-3 PUFA ratio decreased from 1.48 to 8.35 times, in the thigh muscles from 1.32 to 5.15 times, and in the liver from 1.32–3.29 times, in comparison with control diets. Enrichment of poultry diets with camelina seeds, cake and oil resulted in such fatty acid profile changes in poultry tissues, which allowed the production and supply of healthier poultry to consumers. According to European Commission Nutrition Claims [117], chicken breast and liver produced with, respectively 16–24% and 10% dietary camelina cake, can be labeled as “high in omega-3 fatty acids”, because the EPA and DHA content was higher than 80 mg/100 g [7,97]. Meanwhile, the EPA and DHA content in the thigh and breast tissues of chickens fed, respectively, 16–24% and 8% cake, was higher than 40 mg/100 g, and these products could be labeled as “a source of omega-3 fatty acids” [97]. The above results might arouse consumer interest and, consequently, lead to higher consumption of valuable n-3 LC PUFA. 

## 9. Conclusions

Camelina seed and its by-product from oil or biodiesel production, such as cake, can be used for meat poultry feeding because they are a valuable feed rich in crude protein (25–40%), oil (6–37%) and antioxidant substances. The content of crude protein and composition of camelina amino acids is close to that of rapeseed meal. 

Camelina is distinguished by a unique fatty acid composition, as ALA accounts for 25.88 to 36.67% of the total fatty acids.

However, camelina also contains antinutrients, especially glucosinolates, that prevent the use of seed and its by-products in poultry nutrition on a larger scale. 

Addition of camelina seed, oil, cake to poultry diets results in 1.32 to 7.23 times higher ALA content in chicken muscles, in comparison with conventional chicken diets. Consequently, higher ALA content reduces the n-6/n-3 PUFA ratio from 1.32 to 8.35 times in muscles. 

Poultry with a higher n-3 PUFA content is beneficial to consumers in their pursuit of healthier products, as such meat increases the consumption of currently deficient n-3 PUFAs and consequently lowers the risks of cardiovascular diseases. Moreover, the use of camelina cake in poultry diets lowers the cost price of poultry and enhances the sustainability of poultry growing and biofuel production. A wider use of camelina should reduce, at least partly, the dependence on imported non-sustainable soya bean meal, and induce its cultivation worldwide, thus, increasing the crop variety used in agriculture.

## Figures and Tables

**Table 1:** The chemical composition of camelina seed and by-products (as-fed basis).

Parameters	Seed		Cake						
					References				
	[15]	[32]		[33]		[34]	[8]	[35]	[36]
Metabolizable energy, MJ kg^−1^	14.13	17.88		-		9.11	-	-	-
Dry matter, %	93.66			92.02		95.81	93.45	91.95–92.10	90.89
Crude protein, %	24.78	37.17		34.99		36.88	33.31	34.25–34.40	39.8
Ether extract, %	36.84	19.17		13.55		6.44	16.91	21.00–22.71	12.7
Crude fiber, %	11.40	10.72		9.90		17.40	10.53	9.37	12.0
Crude ash, %	4.27	6.80		5.67		5.97	4.91	5.01–5.38	6.30
Neutral-detergent fiber, %	-	35.63		-		45.50	-	26.32–26.63	38.30

**Table 2:** Amino acid composition of camelina cake (as-fed basis).

Amino Acids, %	References					
	[33]	[32]	[46]	[8]	[47]	[36]
Essential amino acids						
Arginine	2.86	2.39	2.64	2.57	2.68	2.87
Histidine	0.83	0.67	0.78	0.73	0.80	0.85
Glycine	1.77	1.66	-	1.59	1.68	1.81
Isoleucine	1.25	1.33	1.11	1.10	1.22	1.34
Leucine	2.20	2.04	2.26	2.00	2.16	2.33
Lysine	1.59	1.25	1.62	1.47	1.52	1.72
Methionine	0.59	0.56	1.64 *	0.57	0.58	0.64
Phenylalanine	1.44	1.44	1.37	1.31	1.39	1.44
Proline	1.77	1.53	-	1.52	1.77	1.75
Threonine	1.34	1.24	1.56	1.23	1.33	1.42
Valine	1.75	1.68	2.11	1.74	1.66	1.80
Tryptophan	-	0.41	-	-	0.41	0.41
Conditionally essential amino acids						
Cystine	0.74	0.65	-	0.79	0.71	0.74
Tyrosine	-	0.87	-	0.91	-	0.92
Nonessential amino acids						
Alanine	1.52	1.44	-	1.31	1.50	1.55
Aspartic acid	2.83	2.62	-	2.31	2.74	2.87
Glutamic acid	5.74	5.20	-	4.99	5.47	5.79
Serine	1.51	1.21	-	1.38	1.51	1.43

* Methionine + cysteine.

**Table 3:** Fatty acid composition of camelina seed, oil, cake, meal (% of total fatty acids).

Fatty Acid	Seed	Oil	Cake					
	References							
	[15]	[37]	[53]	[52]	[35]	[34]		
Myristic (C14:0)	-	-	0.15	0.11	0.08	0.26		
Pentadecanoic (C15:0)	-	-	-	0.04	-	-		
Palmitic (C16:0)	6.07	5.24	7.43	7.05	6.28–6.44	7.73		
Margaric (C17:0)	-	-	-	0.06	-	-		
Stearic (C18:0)	1.91	2.60	2.01	2.37	2.37–2.68	2.76		
Arachidic (C20:0)	-	-	-	1.51	1.33–1.39	0.99		
Heneicosanoic (C21:0)	-	-	-	0.02	-	-		
Behenic (C22:0)	-	-	-	0.36	0.30–0.31	2.18		
Lignoceric (C24:0)	-	-	-	0.21	-	2.55		
SFA	-	7.84	9.59	11.73	-	-		
Palmitoleic (C16:1n-7)	-	-	0.24	0.22	0.02–0.16	-		
Hexadecenoic (C16:1n-9)	-	-	-	0.08	-	-		
Heptadecenoic (C17:1n-9)	-	-	-	0.05	-	-		
Vaccenic (C18:1n-7)	-	-	-	1.35	-	-		
Oleic (C18:1n-9)	16.46	15.70	17.69	17.11	15.28–17.17	12.8		
Eicosenoic (C20:1n-9)	12.99	14.61	-	12.28	14.04–15.34	8.85		
Erucic (C22:1n-9)	5.02	2.04	-	3.20	2.38	2.31		
Nervonic (C24:1n-9)	-	-	-	0.92	-	-		
MUFA	-	20.62	17.93	35.21	-	-		
Linoleic (C18:2n-6)	18.84	-	21.09	24.16	21.13–22.63	23.47		
Linolelaidic (C18:2n-6 trans)	-	-	-	0.02	-	-		
Octadecadienoic (C18:2n-6cis, trans)	-	-	-	0.04	-	-		
γ—linolenic (C18:3n-6)	-	-	-	0.11	0.24–0.25	-		
α—linolenic (C18:3n-3)	33.43	36.67	29.47	25.88	27.73–28.82	36.11		
Octadecatetraenoic (C18:4n-3)	0.36	-	-	-	-	-		
Eicosadienoic (C20:2n-6)	1.47	1.97	-	1.65	-	-		
Eicosatrienoic (C20:3n-3)	-	-	-	0.84	0.98–1.17	-		
Eicosatrienoic (C20:3n-6)	-	1.48	-	0.00	-	-		
Arachidonic (C20:4n-6)	1.02	-	-	0.05	2.47	-		
Eicosapentaenoic (C20:5n-3)	0.12	-	-	0.00	0.08–0.09	-		
Docosadienoic (C22:2n-6)	-	-	-	0.30	-	-		
Docosatetranoic (C22:4n-6)	0.33	-	-	0.03	-	-		
Docosapentaenoic (C22:5n-3)	0.04	-	-	-	-	-		
Docosahexaenoic (C22:6n-3)	0.34	-	-	-	-	-		
n-6 PUFA	21.66	-	-	26.36	-	-		
n-3 PUFA	34.29	-	-	26.72	-	-		
PUFA/SFA	-	-	-	4.53	-	-		
n-6/n-3	-	0.60	-	0.99	-	-		
Linoleic/α-linolenic	-	-	0.72	-	-	-		

SFA: saturated fatty acids; MUFA: monounsaturated fatty acids; PUFA: polyunsaturated fatty acids.

**Table 4:** Effects of camelina on poultry growth performance.

Poultry/Feed	Level, %	Trial Period, Days	Body Weight, g	Weight Gain, g	Feed Intake, g/Birds	Feed Conversion Ratio, kg/kg	Bird Mortality, %	Reference
Chicken/cake	2.5	1–42	−172.29 *	−173.59 *	+2.3	+0.17	-	[7]
	5		+54.37	+54.43	+312.8	+0.11	-	
	10		−59.69	−59.01	+182.1	+0.15	-	
Chicken/oil, cake	Oil, 4	22–42	-	−22	−50	−0.04	+0.66	[85]
	Cake, 10		-	−122	−116	+0.09	+0.75	
Chicken/oil, seed	Oil, 2.5	11–42	+63.82	-	+87.8	−0.01	−0.38	[15]
	Seed, 5		−31.86	-	+134.85	+0.08	+0.39	
	Seed, 10		−188.29 *	-	−40.44	+0.13	+0.19	
Quail/cake	5	1–35	+2.65	+2.65	+7.71	−0.00001	-	[34]
	10		+1.06	+0.94	+10.9	+0.00004	-	
	15		+3.35	+2.38	+31.27	+0.00013 *	-	
	20		−2.31	−3.53	+17.92	+0.00017 *	-	
Turkey/cake	5	1–28	−32	-	+19	+0.09		[86]
	15		−56	-	+4	+0.12	-	
	5	1–28	−66	-	−90	+0.03	-	[86]
	15		−154 *	-	−226 *	+0.06	-	
	20		−216 *	-	−197 *	+0.30 *	-	
Chicken/oil	6.91 4.07	1–21 22–35	-	−10	+50	+0.03	-	[37]
			In comparison with soybean oil					
			-	−70	−170	−0.03	-	
			In comparison with rapeseed oil					
Chicken/seed	10	7–42	−116.8 *	−122.13 *	−250	+0.01	+0.5	[87]
Chicken/oil	3	22–49	+61	-	-	−0.03	+0.09	[88]
	6		+76	-	-	−0.04	+2.62	
Chicken/cake	10	1–21	−60 *	-	−71 *	+ 0.03	-	[33]
Chicken/cake	8	23–42	−35.69	−22.17	−17.5	+ 0.01	-	[53]
Chicken/cake	10	1–42		+107.5	+3.13	+0.10	-	[89]
Chicken/cake	5 male	1–37	−215 *	-	-	-	-	[8]
	5 female		−67	-	-	-	-	
	10 male		−264 *	-	-	-	-	
	10 female		−128 *	-	-	-	-	
	5	1–14	-	-	−3 * per day	-	-	
	10		-	-	−4.3 * per day	-	-	
	5	15–37	-	-	−3 per day	-	-	
	10		-	-	−5 per day	-	-	
	5	1–37	-	-	-	+0.05 *	-	
	10		-	-	-	+0.08 *	-	
Chicken/cake	8	1–42	+334.5 *	+8 *	+1.7	-	+0.54	[35]
	16		+508.5 *	+12.2 *	+0.7	-	+11.29 *	
	24		+105.6	+2.6 *	−1	-	+12.89 *	

Numbers in columns indicate the difference with control group; significant difference found. * *p* < 0.05.

**Table 5:** Effects of camelina on anatomical dissection data.

Poultry/Feed	Level%	Trial Days	CY, %	BM, %	LM, %	AF, %	L, %	H, %	G, %	Lymphoid Tissue, %			Reference
										S	T	BF	
Chickenoil, seed	Oil, 2.5	11–42	+0.4	+2	+3	−0.09	-	-	-	+0.01	−0.02	−0.02	[15] ^1^
	Seed, 5		−0.2	−2	−4	−0.14	-	-	-	−0.02	−0.03	+0.02	
	Seed, 10		−0.7	−4	−7	−0.52 *	-	-	-	−0.01	−0.06	−0.11 *	
Quail/ cake	5	1–35	-	-	-	-	−0.31	−0.08	-	−0.02	-	-	[34]
	10		-	-	-	-	−0.22	−0.07	+0.05	−0.01	-	-	
	15		-	-	-	-	−0.23	−0.03	+0.08	0	-	-	
	20		-	-	-	-	−0.38	−0.05	+0.12	−0.01	-	-	
Chicken/cake	2.5	1–42	-	-	-	+0.46	+0.1	−0.08	+0.12	−0.01	-	-	[7]
	5		-	-	-	+0.41	−0.37	−0.04	-	+0.01	-	-	
	10		-	-	-	+0.45	−0.40	−0.11	-	0	-	-	
Chicken/oil, cake	Oil, 4	22–42	+0.74	+0.44	+0.42	−0.09	−0.09	-	-	-	-	-	[85]
	Cake, 10		+0.95	−2.12 *	+0.23	−0.15	−0.09	-	-	-	-	-	
Chicken/ oil	3	22–49	−0.8	−0.4	−0.2	−0.10	−0.10	-	-	-	-	-	[88]
	6		−0.4	−0.3	−0.1	−0.02	−0.11	-	-	-	-	-	
Chicken/cake	10	1–21	+0.1	0	0	-	-	-	-	-	-	-	[89]
Chicken/ cake	8	23–42	-	-	-	−0.1	-	-	-	−0.02	−0.03	−0.05 *	[53]
Chicken/ cake	8	1–42	-	-	-	-	+1.29	+0.05	-	+0.02	-		[35] ^2^
	16		-	-	-	-	−0.97	+0.29	-	+0.13	-	-	
	24		-	-	-	-	0	+0.29	-	+0.10	-	-	

CY: carcass yield; BM: breast muscles; LM: leg muscles; AF: abdominal fat; L: liver; H: heart; G: gizzard; S: spleen; T: Thymus; BF: Bursa of Fabricius. Numbers in columns indicate the difference with control group; significant difference found. * *p* < 0.05. ^1^ Differences of breast and leg muscles weight in grams. ^2^ Differences of organ weight g/kg BW.

**Table 6:** Effects of camelina on chemical composition of chicken breast muscles.

Poultry/ Feed	Trial Period, Days	Level, %	Parameters			Reference
			Dry Matter, %	Protein, %	Fat, %	
Chicken/ oil, cake	22–42	Oil, 4	−0.47	−0.42	+0.04	[85]
		Cake, 10	+0.18	−0.08	+0.09	
Chicken/ oil	22–49	3	+0.46	+0.47 *	+0.04	[88]
		6	+0.32	+0.41	+0.04	
Chicken/ oil, seed	11–42	Oil, 2.5	+0.04	+0.25	+0.26	[15]
		Seed, 5	+0.29	+0.73 *	+0.32	
		Seed, 10	−0.43	+1.62 *	+0.37	
Chicken/ cake	1–42	2.5	-	-	+0.12	[7]
		5	-	-	+0.07	
		10	-	-	+0.41	

Numbers in columns indicate the difference with control group; significant difference found. * *p* < 0.05.

**Table 7:** The results of blood plasma analysis in broiler chicken.

Poultry/ Feed	Level, %	Trial Days	Glucose, mg/dL	Cholesterol, mg/dL	HDL, mg/dL	LDL, mg/dL	LDL/ HDL	Triglycerides, mg/dL	Reference
Chicken/cake	8	23–42	−11.84 *	−13.31 *	−6.66 *	−7.72 *	-	−4.61	[53]
Chicken/oil, seed	Oil, 2.5	11–42	+8.7	−18.1 *	−7.9 *	−2.3	-	−0.7	[15]
	Seed, 5		+1.6	−14.4 *	−5.5 *	−6.9	-	+0.2	
	Seed, 10		+4.9	−25.7 *	−22.4 *	−8.3 *	-	+2.8	
Chicken/oil	3	22–49	-	−3	−1.3	−2.8 *	−0.03	+5.8	[88]
	6		-	−21.7 *	−15.4 *	−8 *	−0.05	+8.5	

HDL: High Density Lipoprotein; LDL: Low Density Lipoprotein. Numbers in columns indicate the difference with control group; significant difference found * *p* < 0.05.

**Table 8:** Effects of camelina on SFA and MUFA composition in breast, leg meat and liver.

Poultry Feed	Level, %	Trial, Days	C14:0	C16:0	SFA	C18:1	MUFA	Reference
**Breast**								
Chicken cake	8	1–42	0	−0.5	−1.1	−2.7 *	−2.8 *	[97]
	16		+0.02	−1.5 *	−2.2 *	−5.1 *	−5.7 *	
	24		−0.02	−3 *	−2.8 *	−7.3 *	−8.5 *	
Chicken cake	2.5	1–42	-	−0.34	+0.01	+0.59	+0.71	[7]
	5		-	−0.56	−0.84	+0.30	+0.29	
	10		-	+0.95	−0.41	+5.37 *	+6.82 *	
Chicken oil, cake	Oil, 4	22–42	-	+1.45	+1.95	−6.66 *	−6.36 *	[85]
	Cake, 10		-	−0.38	−1.16	−3.29 *	−3.23 *	
Chicken oil, seed	Oil, 2.5	11–42	-	−0.24	-	+3.18 *	-	[15]
	Seed, 5		-	−0.42	-	+2.62 *	-	
	Seed, 10		-	−0.13	-	+2.89 *	-	
Chicken cake	5	1–38	-	−0.5 *	-	−3.2 *	-	[98]
	10		-	−2.0 *	-	−4 *	-	
	15		-	−2.2 *	-	−5.6 *	-	
	20		-	−2.5 *	-	−6.2 *	-	
	25		-	−2.2 *	-	−10.2 *	-	
Duck cake	15–20	1–49	+0.02	+0.53	+0.51	−2.89	−2.25	[52]
Chicken oil	6.91 4.07	1–21 22–35	-	+1.39	+1.08	+16.46 *	-	[37]
			In comparison with soybean oil					
			-	+5.71 *	+6.41 *	−14.97 *	-	
			In comparison with rapeseed oil					
**Leg**								
Chicken cake	8	1–42	0	+0.9	+1.0	−1.4	−1.7	[97]
	16		0	−4.0	−3.2	−2	−4.1	
	24		−0.1	−4.4	−3.9	−4.2	−6.9 *	
Chicken cake	2.5	1–42	+0.02	−0.30	+1.33	−1.41	−1.49	[7]
	5		+0.01	+0.47	+1.76	−1.19	−1.15	
	10		+0.01	+0.60	+1.97	−1.34	−0.66	
Chicken cake female	5	1–37	-	−0.67	−0.31	−1.25	−0.90	[8]
	10		-	−3.21 *	−2.42 *	−3.51 *	−2.29 *	
Chicken cake male	5	1–37	-	−1.24 *	−1.39 *	−1.98	−1.58 *	
	10		-	−3.12 *	−3.73 *	−3.56 *	−2.21 *	
Duck cake	15–20	1–49	+0.03	+1.37 *	+2.25 *	−4.85 *	−4.10 *	[52]
Chicken oil	6.91 4.07	1–21 22–35	-	−1.98 *	−1.77	+5.48	-	[37]
			In comparison with soybean oil					
			-	+2.84 *	+5.22 *	−21.8 *	-	
			In comparison with rapeseed oil					
**Liver**								
Chicken cake	8	1–42	+0.01	0	+3.1 *	−6 *	−6.7 *	[97]
	16		−0.06 *	−2.1 *	+4.4 *	−12.3 *	−14.1 *	
	24		−0.14 *	−6.0 *	+4.3 *	−19.9 *	−22.7 *	
Chicken cake	2.5	1–42	−0.29 *	+0.16	−8.26 *	−3.27	−3.44	[7]
	5		−0.25 *	+0.54	+4.98 *	−4.47	−4.92	
	10		−0.12	−0.02	+2.25	−4.52	−4.75	

Numbers in columns indicate the difference with control group; significant difference found. * *p* < 0.05.

**Table 9:** Effects of camelina on PUFA composition in breast, leg meat and liver.

Poultry/ Feed	Level,%	Trial Days	C18:3	C20:3	C20:5	C22:5	C22: 6n-3	LC n-3 PUFA	n-3 PUFA	C18: 2n-6	n-6 PUFA	n-6/n-3 PUFA	Reference
**Breast**													
Chicken/ cake	8	1–42	+2.1 *	+0.12 *	+0.01	+0.26	0	+0.3	+2.3 *	+1.6 *	+1.4	−1.2 *	[97]
	16		+3.4 *	+0.33 *	−0.03	+0.58 *	+0.1	+1 *	+4.3 *	+3.4 *	+3.4 *	−1.5 *	
	24		+4.8 *	+0.37 *	0	+0.64 *	+0.1	+1.1 *	+5.7 *	+5.1 *	+5.4 *	−1.6 *	
Chicken/ cake	2.5	1–42	+0.59 *	-	+0.03	+0.21	+0.34	-	+1.16 *	+0.48	−1.88	−6.4 *	[7]
	5		+0.95 *	-	+0.08	+0.33	+0.28	-	+1.62 *	+1.85	−1.07	−7.62 *	
	10		+1.62 *	-	+0.14	+0.12	+0.22	-	+2.10 *	−2.37	−8.5 *	−10.44 *	
Chicken/ oil, cake	Oil, 4	22–42	+4.5 *	-	-	-	-	-	+4.88 *	+0.16	−0.68	−2.04 *	[85]
	Cake, 10		+3.69 *	-	-	-	-	-	+3.73 *	+1.49 *	+0.51	−1.66 *	
Chicken/ oil, seed	Oil, 2.5	11–42	+2.46 *	-	+0.42 *	+0.19 *	+0.48 *	-	+3.50 *	+0.83	−2.36 *	-	[15]
	Seed, 5		+1.73 *	-	+0.15 *	+0.16 *	+0.40 *	-	+2.37 *	+1.30	−1.47 *	-	
	Seed, 10		+2.38 *	-	+0.55 *	+0.25 *	+0.46 *	-	+3.55 *	+0.59	−2.41 *	-	
Chicken/ cake	5	1–38	+1.72 *	-	+0.32 *	-	+0.54 *	-	+3.36 *	+1	+1.3 *	−2.24 *	[98]
	10		+3.63 *	-	+0.52 *	-	+0.36 *	-	+5.33 *	+1	+1.0 *	−2.23 *	
	15		+3.56 *	-	+0.54 *	-	+0.76 *	-	+6.03 *	+1.5	+2.3 *	−2.33 *	
	20		+4.72 *	-	+1.01 *	-	+0.86 *	-	+8.33 *	0	+0.7 *	−2.38 *	
	25		+5.07 *	-	+1.08 *	-	+1.78 *	--	+10.43 *	+2	+3.4 *	−2.88 *	
Duck/ cake	15–20	1–49	+1.49 *	+0.08 *	+0.08	+0.09	+0.13		+1.86 *	+0.28	+0.01	−2.16 *	[52]
Chicken/ oil	6.91 4.07	1–21 22–35	+3.07 *	-	-	-	-	-	-	−21.27 *	-	−35.14 *	[37]
			In comparison with soybean oil										
			+3.55 *	-	-	-	-	-	-	+4.13 *	-	−17.82 *	
			In comparison with rapeseed oil										
**Leg**													
Chicken/cake	8	1–42	+1.7 *	+0.08 *	−0.01	+0.18 *	+0.07	+0.33	+2.1 *	−0.9	−1.4	−3 *	[97]
	16		+4.2 *	+0.20 *	0	+0.21 *	+0.06	+0.47 *	+4.7 *	+2.5	+2.6	−2.9 *	
	24		+6.4 *	+0.23 *	0	+0.27 *	+0.14	+0.63 *	+7.1 *	+3.9 *	+3.6	−3.6 *	
Chicken/cake	2.5	1–42	+0.37	-	+0.08	+0.37 *	+0.38 *	-	+1.2 *	−2.44	−1.04	−9.38 *	[7]
	5		+0.61 *	-	+0.04	+0.33 *	+0.25 *	-	+1.23 *	−2.58	−1.84	−9.83 *	
	10		+1.45 *	-	+0.23 *	+0.42 *	+0.40 *	-	+2.5 *	−3.93	−3.81	−13.91 *	
Chicken cake female	5	1–37	+1.48 *	-	-	-	-	-	+1.55 *	−0.16	+0.04	−1.11 *	[8]
	10		+4.02 *	-	-	-	-	-	+4.17 *	+1.79 *	+2.16 *	−1.89 *	
Chicken cake male	5	1–37	+1.88 *	-	-	-	-	-	+2.05 *	+0.62	+0.68	−1.46 *	
	10		+4.53 *	-	-	-	-	-	+4.69 *	+1.63 *	+1.56	−2.39 *	
Duck/ cake	15–20	1–49	+2.33 *	+0.10 *	+0.06	+0.04	+0.06	-	+2.5 *	+0.29	+0.02	−2.94 *	[52]
Chicken/ oil	6.91 4.07	1–21 22–35	+7.59 *	-	-	-	-	-	-	−7.90 *	-	−11.57 *	[37]
			In comparison with soybean oil										
			+8.45 *	-	-	-	-	-	-	+10.44 *	-	−9.43 *	
			In comparison with rapeseed oil										
**Liver**													
Chicken/ cake	8	1–42	+0.47 *	+0.08 *	+0.02	+0.21 *	+0.50 *	+0.8 *	+1.3 *	+2.2 *	+2.3 *	−1.2 *	[97]
	16		+1.13 *	+0.19 *	+0.03	+0.59 *	+1.24 *	+2.0 *	+3.2 *	+4.8 *	+6.5 *	−1.7 *	
	24		+2.10 *	+0.37 *	+0.03	+0.97 *	+2.63 *	+3.9 *	+6.1 *	+9 *	+12.4 *	−2.2 *	
Chicken /cake	2.5	1–42	−0.25	-	+0.21 *	+0.80 *	0	-	+2.58 *	−7.91 *	−2.83	−6.45 *	[7]
	5		−0.21	-	+0.31 *	+0.64	+3.05 *	-	+3.79 *	−7.77 *	−3.86 *	−7.66 *	
	10		+0.43	-	+0.45 *	+1.19 *	+4.02 *	-	+6.09 *	−5.01	−3.59 *	−8.85 *	

Numbers in columns indicate the difference with control group; significant difference found. * *p* < 0.05.

## Data Availability

Data are available from the Corresponding author on request.
